# Dermoscopic features of cutaneous endometriosis arising in a caesarean section^؆^

**DOI:** 10.1016/j.abd.2026.501440

**Published:** 2026-08-06

**Authors:** Şule Yıldız Sağcan Tercan, Ergün Tercan, Peyker Temiz, Can Tercan, Muhammet Canbulut, Cemal Bilaç

**Affiliations:** a Department of Dermatology and Venereology, Manisa Celal Bayar University, Manisa, Turkey; b Manisa Merkezefendi State Hospital, Manisa, Turkey; c Department of Pathology, Manisa Celal Bayar University, Manisa, Turkey; d Başaksehir Çam and Sakura City Hospital, Istanbul, Turkey

Dear Editor,

Endometriosis is characterized by the presence and growth of endometrial glandular and/or stromal tissue outside the uterine cavity and most commonly involves pelvic structures; however, extrapelvic manifestations have also been described.^1^

Herein, we present a case of cutaneous endometriosis developing within a caesarean section scar and highlight its distinctive dermoscopic characteristics.

A 29-year-old female with Fitzpatrick skin type III presented to our dermatology clinic with a tumorous growth arising within a caesarean section scar. The patient was otherwise healthy and had no history of dermatological disease. She had undergone two caesarean sections, the most recent one four years earlier, without postoperative complications. Two years after the last procedure, she noticed the development of a small, painful nodular lesion located at the midline of the surgical scar, which progressively enlarged over a six-month period.

On clinical examination, the lesion measured approximately 1.5 × 2 cm and appeared as a well-circumscribed, firm nodular mass with a slightly elevated and irregular surface, showing a bluish-gray discoloration within the caesarean scar (Fig. 1). Dermoscopic examination (Fig. 2) revealed a predominantly structureless brownish to pale background containing multiple violaceous to dark red clods of variable size. Numerous dotted vessels were observed, some surrounded by whitish structureless areas, resulting in a subtle reticular arrangement. The lesion exhibited a flat surface without papillomatous projections. Scattered pale structureless zones and minimal surface scaling were present. No pigment network or other dermoscopic criteria suggestive of a melanocytic lesion were identified. Upon further questioning, the patient reported cyclical bleeding from the lesion in correlation with her menstrual cycle.

**Fig. 1:**
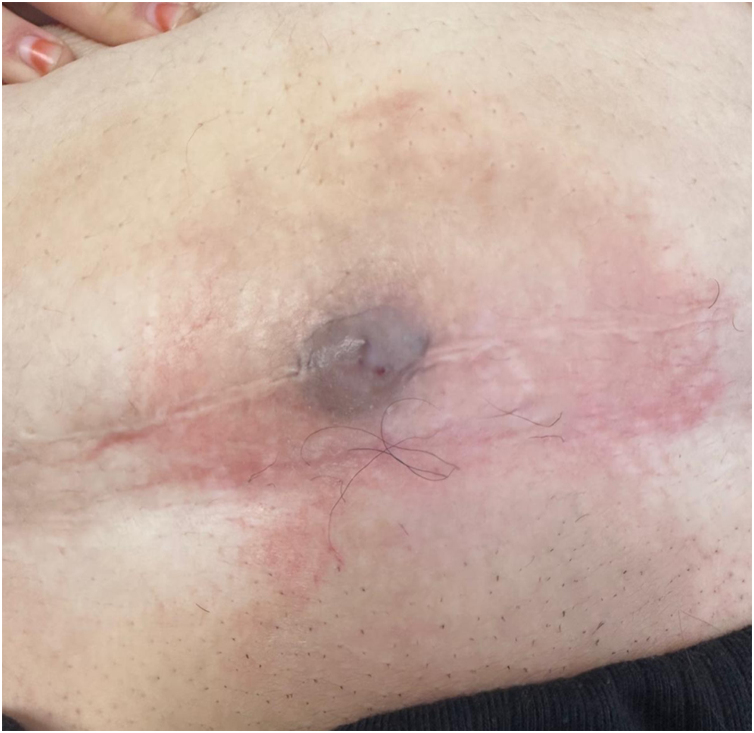
Clinical photograph showing a well-circumscribed, bluish-gray nodular lesion arising at the midline of the caesarean section scar.

**Fig. 2:**
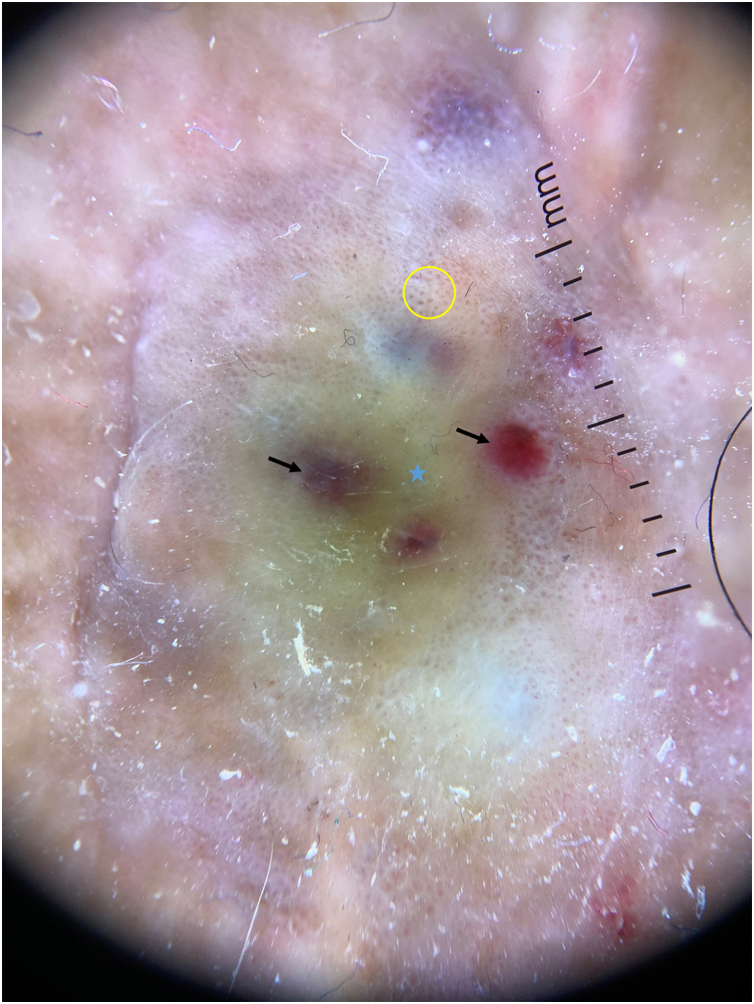
Dermoscopic image showing a brownish structureless background (blue star) with violaceous to dark red clods (black arrow), predominantly dotted vessels, and whitish structureless areas (yellow circle) (polarized, 10×).

An excisional biopsy was performed, and histopathological examination confirmed the diagnosis of cutaneous endometriosis (Fig. 3). Ultrasonographic and magnetic resonance imaging evaluations revealed no additional pathology apart from the described lesion. The patient was referred to the gynecology department and successfully treated with complete surgical excision.

**Fig. 3:**
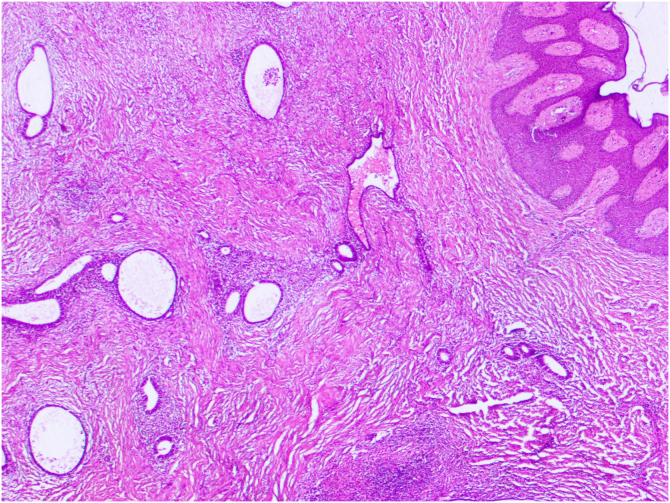
Endometrial glands surrounded by endometrial stroma within the dermis (Hematoxylin & eosin, ×400).

Cutaneous endometriosis is defined by the ectopic implantation of endometrial glandular and/or stromal tissue within the skin and represents a rare manifestation, accounting for less than 1% of all ectopic endometriosis sites. It is conventionally classified into primary cutaneous endometriosis, which develops in the absence of prior surgical procedures, and secondary cutaneous endometriosis, most often arising in association with previous abdominal surgery.^2^ Surgical scar endometriosis occurring after Caesarean Section (C/S) has been reported with increasing frequency in recent years, a trend that is largely attributed to the rising number of caesarean deliveries.^3^

Intraoperative contamination is recognized as a major contributor to the pathogenesis of cesarean scar endometriosis, particularly when materials utilized during uterine closure are subsequently employed for abdominal wall layers, enabling the transfer of endometrial cells.^4^ Preventive strategies primarily involve maintaining strict separation of surgical materials between tissue planes, avoiding instrument reuse, and performing thorough irrigation of the operative field prior to closure.^5^, ^6^

In this context, endometriosis arising in a C/S scar should be considered in the differential diagnosis of surgical scar lesions, including granuloma, hematoma, incisional hernia, keloid formation, and malignancy.^2^, ^7^

Dermoscopy provides additional supportive information in the assessment of cutaneous endometriosis, although the observed dermoscopic features may vary depending on factors such as lesion location, depth, histological characteristics, and patient phototype, which can complicate the recognition of a consistent dermoscopic pattern.^8^

Previously reported dermoscopic findings of cutaneous endometriosis arising in a caesarean section scar are limited. Tognetti et al.^9^ described pigmented arborizing lines, granular structures on a gray background, bright dots, and targetoid brownish elements, none of which were observed in our case. Similarly, the lesion reported by Jaime et al., characterized by homogeneous structureless reddish pigmentation and amorphous brown areas, differed from our findings both clinically and dermoscopically.^10^

Although Costa et al.^8^ reported erythematous–violaceous polypoid projections with light brown round spots and cyclic changes related to the menstrual phase, our lesion lacked papillomatous projections and instead demonstrated multiple violaceous to dark red clods over a predominantly structureless brownish to pale background. Accordingly, numerous dotted vessels were observed, some partially surrounded by whitish structureless areas, resulting in a subtle reticular arrangement, which appears to be consistent with the pattern described by Yang et al.^11^ characterized primarily by dotted vessels encompassed by a white reticular network.

This case report contributes to the limited existing literature on the dermoscopic features of cutaneous endometriosis and further underscores the rarity of this condition.

## CRediT authorship contribution statement

Şule Yıldız Sağcan Tercan: Approval of the final version of the manuscript; critical literature review, analysis and interpretation; effective participation in research orientation; manuscript critical review, preparation and writing of the manuscript.

Ergün Tercan: Approval of the final version of the manuscript; manuscript critical review, preparation and writing of the manuscript.

Peyker Temiz: Approval of the final version of the manuscript; manuscript critical review.

Can Tercan: Approval of the final version of the manuscript; manuscript critical review.

Muhammet Canbulut: Approval of the final version of the manuscript; manuscript critical review.

Cemal Bilaç: Approval of the final version of the manuscript; manuscript critical review.

## Financial support

None declared.

## Research data availability

Does not apply.

## Declaration of competing interest

None declared.
